# Mesenteric Route Superior Mesenteric Artery First Approach in Robot-Assisted Pancreatoduodenectomy

**DOI:** 10.1245/s10434-025-18087-x

**Published:** 2025-08-18

**Authors:** Kosei Takagi, Atene Ito, Tomokazu Fuji, Kazuya Yasui, Takeyoshi Nishiyama, Tsubasa Yanagihara, Toshiyoshi Fujiwara

**Affiliations:** Department of Gastroenterological Surgery, Okayama University Graduate School of Medicine, Dentistry, and Pharmaceutical Sciences, Okayama, Japan

**Keywords:** Robotic pancreaticoduodenectomy, Superior mesenteric artery approach, Mesenteric route

## Abstract

**Background:**

The superior mesenteric artery (SMA) approach is crucial for the successful implementation of robot-assisted pancreatoduodenectomy (RPD). Herein, we present a novel technique, the mesenteric route SMA-first approach, for RPD.

**Patients and Methods:**

A 20-year-old woman with a 50 mm intraductal papillary mucinous neoplasm underwent RPD. As the tumor was large and located close to the mesenteric vessels, we developed the mesenteric route SMA-first approach.

**Results:**

Following the mesenteric Kocher maneuver, the mesenteric route SMA-first approach was applied. With appropriate retraction of the pancreatic head, dissection around the mesenteric vessels was performed and their branches were divided. The uncinate process dissection (PL, ph II) was performed via the mesenteric route. This approach facilitated dorsal dissection, particularly around the large tumor. After dissection of the hepatoduodenal ligament, the remaining pancreatic nerve plexus (PL ph I) was dissected. Finally, the pancreas was divided on the superior mesenteric vein, and the specimen was resected. Operative time was 390 min with minimal blood loss.

**Conclusions:**

The mesenteric route SMA-first approach enables uncinate process dissection via the mesenteric route. This technique may be a safe and feasible option for selected patients, such as nonobese individuals with a large pancreatic head tumor near major vessels.

**Supplementary Information:**

The online version contains supplementary material available at 10.1245/s10434-025-18087-x.

Several surgical approaches to the superior mesenteric artery (SMA) have been reported in robot-assisted pancreatoduodenectomy (RPD).^1,2^ Selecting the most appropriate SMA approach is essential for successful RPD. Herein, we present our novel mesenteric route SMA-first approach in RPD (supporting video 1).

## Case

A 20-year-old woman with a 50 mm intraductal papillary mucinous neoplasm underwent RPD. Her body mass index was 20.1 kg/m^2^. Preoperative computed tomography revealed a large cystic tumor at the pancreatic head, close to the mesenteric vessels. Operative time was 390 min with minimal blood loss. The patient had an uneventful clinical course and was discharged on postoperative day 13.

## Surgical Technique

**Fig. 1:**
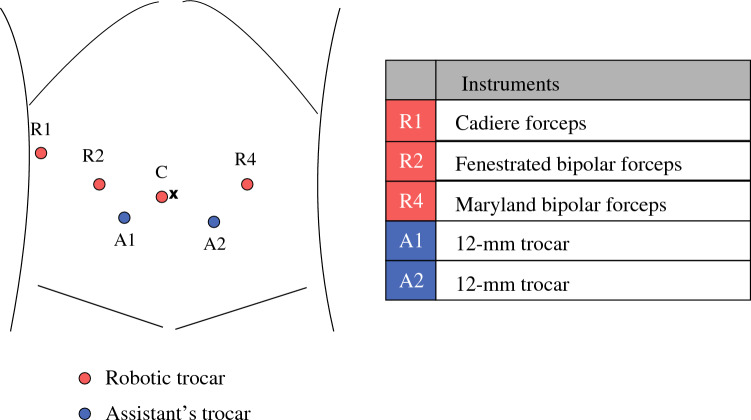
Trocar placement in robot-assisted pancreatoduodenectomy

The da Vinci Xi platform (Intuitive Surgical, Sunnyvale, CA, USA) was used. Patient was placed in the seven-degree reverse Trendelenburg position and seven-degree tilt left. The port placements and instruments are shown in Fig. 1. Initially, the transverse colon was lifted cranially using the robotic arm (Cadiere forceps), and the mesenteric Kocher maneuver was performed to mobilize the pancreatic head via the mesenteric route.^3^ Dissection was performed using the double bipolar method, using the fenestrated bipolar forceps and Maryland bipolar forceps. The jejunum was divided using a stapler and pulled to the right side through the mesentery.

**Fig. 2:**
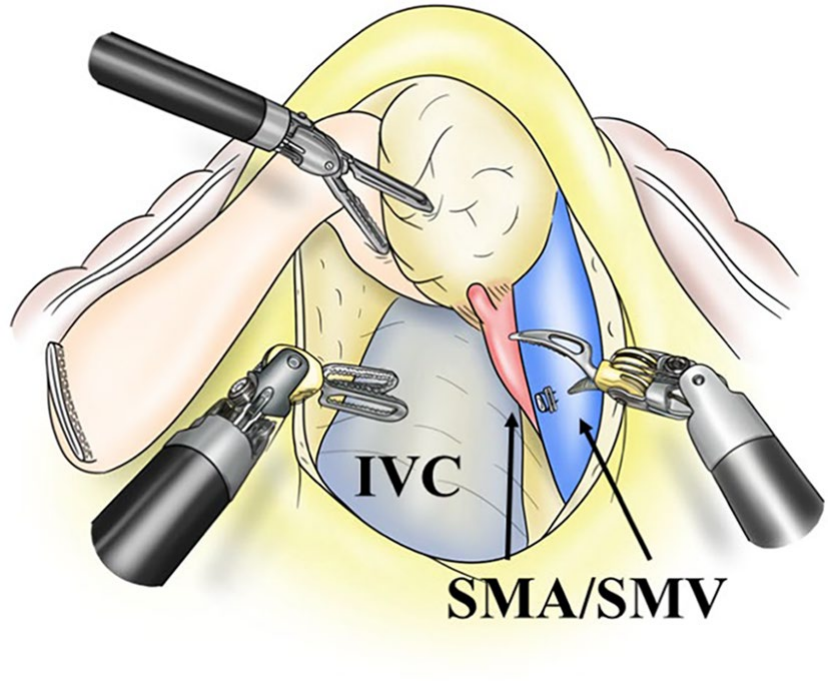
Mesenteric route SMA-first approach in robot-assisted pancreatoduodenectomy; uncinate process dissection can be performed through the mesenteric route; *IVC* inferior vena cava, *SMA* superior mesenteric artery, *SMV* superior mesenteric vein

Subsequently, the mesenteric route SMA-first approach was applied (Fig. 2). With appropriate retraction of the pancreatic head, dissection around the mesenteric vessels was performed and their branches were divided. The uncinate process dissection (PL ph II) was also performed via the mesenteric route. This approach facilitated dissection, particularly on the dorsal side of the large tumor.

After repositioning the transverse colon, the anterior side of the tumor was dissected. The hepatoduodenal ligament, including the division of the gastroduodenal and common bile ducts, was dissected. The pancreatic head was pulled leftward, and the remaining pancreatic nerve plexus (PL ph I) was dissected. Finally, the pancreas was divided on the superior mesenteric vein, and the specimen was resected.

## Discussion

This study presents the mesenteric route SMA-first approach in RPD. This approach may have several advantages. Using the mesenteric route SMA-first approach, dissection around the mesenteric vessels can be performed through the mesenteric route at an early phase in the caudal view during RPD. This approach can be helpful for dissecting the internal and posterior sides of large tumors through the mesenteric route. In other words, this technique enables safe dissection from the root toward the periphery of the mesenteric vessel branches from the dorsal side. Although the surgical view of the mesenteric route SMA appeared similar to that of the posterior approach, a key difference would be the SMA dissection, either the mesenteric route or the supracolic route. Approaching the dorsal side of large tumors remains a challenge; however, the mesenteric route approach using the caudal view may prevent a split injury in cases with large tumors adjacent to major vessels, compared with traditional anterior or posterior approaches. However, this mesenteric SMA approach has some limitations. This approach may be particularly demanding in patients with obesity because of the limited exposure and depth perception in the deep mesenteric plane. Since this was based on our experience with a single case, the technical reproducibility and generalizability of this approach should be confirmed in future studies.

## Conclusions

The mesenteric route SMA-first approach allows for uncinate process dissection through the mesenteric route in selected patients. This approach may be safe and optional for large tumors of the pancreatic head that are close to the major vessels.

## Supplementary Information

Below is the link to the electronic supplementary material.

Supplementary file1 (MP4 272398 KB)
